# Low-Temperature Solution-Based Molybdenum Oxide Memristors

**DOI:** 10.1021/acsaenm.3c00535

**Published:** 2024-01-24

**Authors:** Raquel Azevedo Martins, Emanuel Carlos, Asal Kiazadeh, Rodrigo Martins, Jonas Deuermeier

**Affiliations:** CENIMAT|i3N, Department of Materials Science, School of Science and Technology, NOVA University Lisbon and CEMOP/UNINOVA, 2829-516 Caparica, Portugal

**Keywords:** MoO_3_, solution-based, molybdenum oxide, memristor, self-rectifying

## Abstract

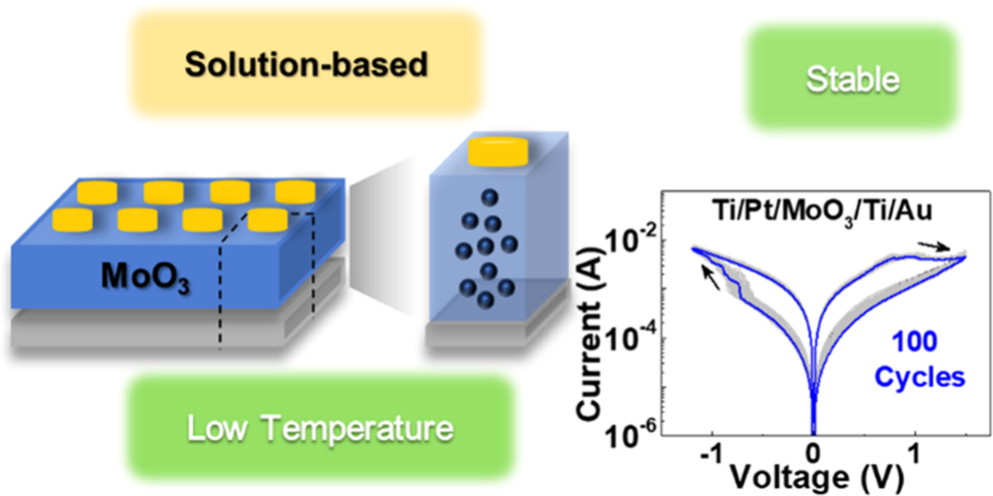

Solution-based memristors have gained significant attention in recent years due to their potential for the low-cost, scalable, and environmentally friendly fabrication of resistive switching devices. This study is focused on the fabrication and characterization of solution-based molybdenum trioxide (MoO_3_) memristors under different annealing temperatures (200 to 400 °C). A MoO_3_ ink recipe is developed using water as the main solvent, enabling a simplified and cost-effective fabrication process. Material analysis reveals the presence of a Mo^6+^ oxidation state and an amorphous structure in the films annealed up to 250 °C. Electrical tests confirm a bipolar resistive switching behavior in the memristors according to the valence change mechanism (VCM). Endurance tests demonstrate stable memristors, indicating their robust nature after multiple cycles. Memristors annealed at 250 °C exhibit a nonvolatile behavior with a retention time up to 10^5^ s under ambient air conditions. The high reproducibility observed in these memristors highlights their potential for practical applications and scalability.

## 1 Introduction

Nonvolatile memory devices (NVMs) are emerging due to the high demand for simultaneous data storage and processing that actual technologies cannot overcome due to physical limitations.^1^ Memristors are one type of NVMs that stand out for their low power consumption, high density, and ability to emulate the synaptic plasticity of the brain,^2^ with an expected market growth of 52% until 2028.^3,4^ One of its major applications is in neuromorphic computing, as memristors can emulate the synaptic behavior of a neuron.^5,6^

Memristors are two terminal devices with fast operation based on a reversible switching (RS) mechanism between a low resistance state (LRS) and a high resistance state (HRS).^7^ These changes in state occur as a result of redox reactions within valence change mechanism (VCM) memristors. When a voltage stimulus is applied to the device, conductive filaments (CFs) are generated and disrupted. The formation of a CF usually involves the movement of oxygen anion species or the electromigration of metal cations within the active layer. The typical structure of VCM memristors includes a transition metal oxide (TMO) between an ohmic-type and a Schottky-type electrode.^8^

Molybdenum trioxide (MoO_3_) is a widely studied TMO. With a wide band gap and a high dielectric constant, MoO_3_ is the most stable state of molybdenum oxide in air.^9^ There are several areas where MoO_3_ has potential applications such as catalysis, sensors, and solar cells.^10−12^ MoO_3_ is also suitable for memristor applications since it has excellent electrical and thermal stability, and it is an abundant noncritical material leading to low-cost green technologies.^7,9^ One of the advantages of this oxide is that its electrical properties can be easily tuned by the manipulation of stoichiometry.^13^ The presence of oxygen vacancies plays a crucial role in determining the conductive behavior of the memristor. Therefore, it becomes vital to have the right amount of oxygen in the memristor in order to achieve the desired performance.

There are a few reports regarding the production of MoO_3_ memristors using vacuum techniques.^9,14^ However, these production methods are expensive and use high temperatures during fabrication.

Solution-based processes allow the production of devices in a simpler, easier, and faster way compared with the common vacuum techniques.^15,16^ However, solution-based memristor devices are novel, and the technology is in an early stage when compared to vacuum-processed production.^7^ The conditions of production are still under optimization once the quality of the films is directly dependent on the manufacturing procedures.

Consequently, there are very few reports studying the potential of solution-based MoO_3_ in memristors. Most of them report memristors of MoO_3_ nanobelts,^17−19^ MoO_3_ mixed with another oxide,^20^ or heterostructures between molybdenum oxide and transition metal dichalcogenides.^21^ Rasool et al. reported a memristor only with MoO_3_ as an active layer deposited by spray pyrolysis using temperatures reaching 400 °C.^13^

In this work, we report a simple methodology to produce solution-based MoO_3_ memristors and study the influence of the annealing temperature as well as the precursor concentration on the device performance in an air environment. All of the memristors show a stable bipolar resistive switching behavior and good endurance up to 100 cycles under the DC voltage sweep. The devices annealed at 250 °C reveal the highest cycling stability and a state retention time of up to 10^5^ s in ambient air.

## 2 Experimental Details

### 2.1 Precursor Solution Synthesis and Characterization

Ammonium molybdate tetrahydrate ((NH_4_)_6_Mo_7_·4H_2_O, Fluka, 99%) was dissolved in deionized (DI) water to produce a metal precursor solution with a concentration of 0.1 M. Under constant stirring, ethylene glycol (EG, C_2_H_6_O_2_, Carlo Erba Reagents, >99.5%) was added to the solution with a proportion of 20% (V/V) to improve the viscosity and conductivity of the films. Sodium lauryl sulfate (SDS, C_12_H_25_NaO_4_S, Scharlau, 95%) was also added to the solution as a dispersant with a proportion of 0.5% (W/V). The precursor solution was stirred for 1 h at room temperature and filtered using a PTFE filter (0.45 μm) before use.

Thermogravimetry and differential scanning calorimetry (TG-DSC) (Netzsch, TG-DSC-STA 449 F3 Jupiter) were performed on the molybdate precursor solution under an air atmosphere up to 550 °C with a 10 °C min^–1^ heating rate in an aluminum crucible.

### 2.2 Thin Film Deposition and Device Fabrication

Metal-insulator-metal (MIM) structures were fabricated on Corning glass substrates. Prior to deposition, all substrates were cleaned as mentioned in a previous report.^16^ The bottom electrode, a Ti/Pt bilayer of 30 and 40 nm, was first deposited on the substrate by e-beam evaporation (homemade apparatus). Then, the MoO_3_ thin films were deposited by spin-coating for 35 s at 3000 rpm (Laurell Technologies), forming a single layer. Each deposition was followed by an immediate hot plate annealing at 200, 250, or 300 °C for 5 min in an air environment (relative humidity (RH): 40–60% at room temperature). This process was repeated twice with a 10 min UV/ozone surface treatment between each deposition. After thin film fabrication, a multilayer of Ti/Au, 6 and 60 nm, respectively, was deposited by e-beam evaporation as the top electrode using a physical mask for patterning (area of 1.96 × 10^–3^ cm^2^). The MoO_3_ thin films were also deposited on glass and p-Si substrates and annealed at different temperatures (200, 250, 300, and 400 °C) for subsequent material characterization.

### 2.3 Thin Film and Device Characterization

The thin film optical properties were obtained using a Shimadzu UV 3101pc UV/vis/NIR spectrophotometer by measuring the transmittance (*T*) in the wavelength range of 250–800 nm.

The structure of the films was assessed by grazing angle X-ray diffraction (XRD), using an X’Pert PRO PANalytical (Royston, U.K.) diffractometer with Cu Kα line radiation (λ = 1.540598 Å) and an incidence angle of the X-ray beam fixed at 0.75°, in the range of 10–90° (2θ).

X-ray photoelectron spectroscopy (XPS) was measured with a Kratos Axis Supra spectrometer. A monochromatic Al Kα source was used, and for the detailed scans, the analyzer was set to pass an energy of 20 eV. Ultraviolet photoelectron spectroscopy (UPS) was performed with the same instrument using He I radiation from a gas discharge lamp. The data was analyzed with CasaXPS software.

The surface morphology of the active layers was investigated by atomic force microscopy (AFM, Asylum MFP3D). Scanning electron microscopy (SEM, Zeiss Auriga Crossbeam microscope) analysis was performed to achieve the MoO_3_ thickness of the sample cross-section. A profilometer (DektakXT) was used to measure the thickness of the MoO_3_ films obtained by annealing at different temperatures after etching a part of the film with phosphoric acid at 85 °C until the film was etched.

The quasi-static current–voltage (*I*–*V*) characteristics and the pulse studies of the devices were measured using a Keithley 4200 SCS semiconductor analyzer connected to a Janis ST-500 probe station. The bias was applied to the top electrode, maintaining the bottom electrode connected to the ground. The speed of the measurements was set to normal mode, and the integration time was in auto setting.

## 3 Results and Discussion

The produced samples have a sandwich structure, where MoO_3_ is the active layer between the Pt bottom and the Ti/Au top electrodes. Material and electrical characterizations performed on the memristors are displayed in this section.

### 3.1 Mo Precursor Solution Characterization

**Figure 1:**
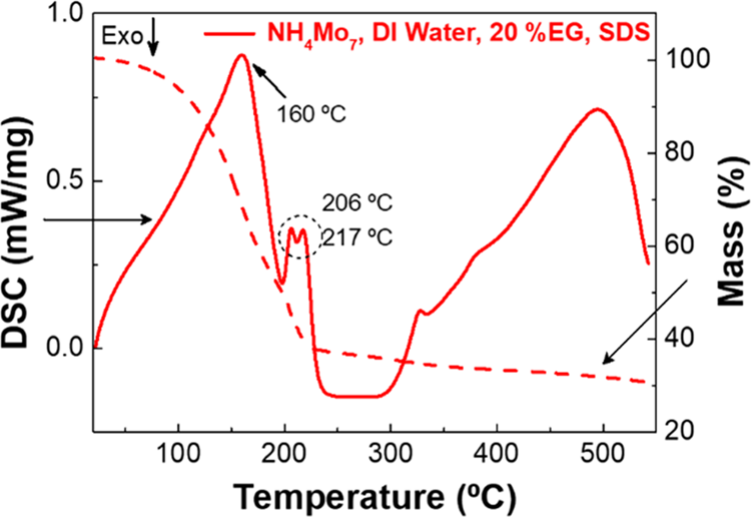
TG-DSC curve of the precursor solution (0.1 M).

Figure 1 shows the TG-DSC curve of the precursor solution of molybdenum with a molar concentration of 0.1 M. Previous to this analysis, the precursor solution was stirred for 5 h at 110 °C on a hot plate to evaporate most of the solvent. The DSC data points reveal three endothermic peaks at 160, 206, and 217 °C. The first peak is related to the loss of water molecules of ammonium molybdate tetrahydrate, accompanied by a mass loss of 50%. The other peaks are due to the boiling of EG added to the precursor solution. After 350 °C, the mass loss is minimal, and only some increment is observed on the DSC analysis being related with the phase changes in the molybdenum oxide crystallinity.^22^ The TG data points confirm that the formation of molybdenum oxide occurs at 250 °C, where the mass loss is minor.

### 3.2 Thin Film Characterization

**Figure 2:**
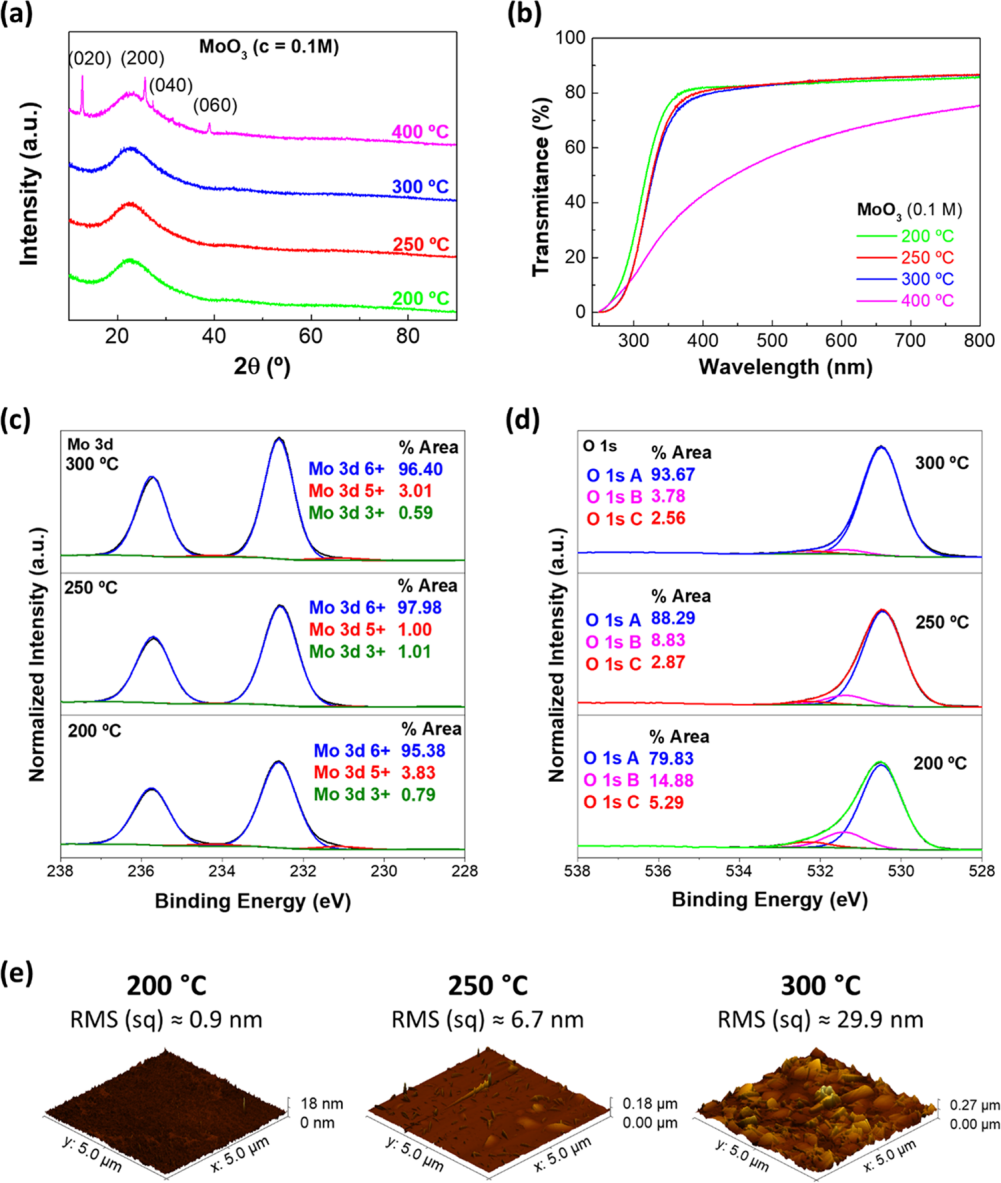
(a) XRD diffractograms, (b) optical transmittance, (c) Mo 3d emission, and (d) O 1s emission of the MoO_3_ thin films annealed at different temperatures. (e) AFM 3D images of the surface roughness in each condition (200, 250, and 300 °C).

The structure of the thin films was analyzed by XRD, as shown in Figure 2a. The results indicate that the thin films annealed below 400 °C have an amorphous structure. At 400 °C, peaks of α-MoO_3_ and β-MoO_3_ phases start to appear; both phases are thermodynamically stable at high temperatures.^23^

The optical transmittance of the MoO_3_ films was measured to observe the transparency of the thin films at different annealing temperatures. The transmittance spectra depicted in Figure 2b indicate that the transparency decreases with the increment of the annealing temperature. Thus, there is an evident decrease of approximately 30% between 300 and 400 °C. Optical band gaps were calculated through the transmittance results from Figure 2b using the Tauc’s plot, as demonstrated in Figure S1.

XPS analysis was performed on the MoO_3_ thin films deposited on platinum. Figure 2c presents the deconvoluted^24^ Mo 3d emission. The main Mo 3d_3/2_ emission at [232.6 ± 0.1] eV corresponds to the Mo^6+^ oxidation state, and the Mo 3d_3/2_ emission at [231.2 ± 0.1] eV corresponds to the Mo^5+^ oxidation state. The lower molybdenum oxidation state decreases from 200 to 250 °C, but no further decrease can be verified for 300 °C. That means basically all films are close to the MoO_3_ stoichiometry, which is supported by the valence band positions with respect to the Fermi level [3.0 ± 0.1] eV and the work functions [5.2 ± 0.1] eV, which remain constant for all samples (Figure S2 and Table S1). Besides the Mo 3d emission, the O 1s spectra have also been fitted (Figure 2d) with three peaks, the main peak (A) related to lattice oxygen and the two subpeaks (B) and (C) typically associated with undercoordinated oxygen, surface oxygen, and/or oxygen of adventitious carbon species as well as surface-adsorbed water. A clear decrease of the relative intensities of subpeaks (B) and (C) with increasing temperature was observed. However, the total carbon concentration shows the same trend, decreasing from 24 to 18% with the increase of temperature. For this reason, the temperature trend of the O 1s subpeaks is mostly related to the decreased hydrocarbon content, rather than undercoordinated or defective oxygen. Since the sulfur concentration also decreases with increasing annealing temperature (from 1.2 to 0.3%), it is concluded that lower annealing temperatures cause more residual precursor components in the films.

To understand the surface roughness of the samples, AFM measurements were performed, as depicted in Figure 2e. These images reveal that the roughness of the surface drastically increases with higher annealing temperatures, changing from near 1 to 30 nm, when the annealing temperature increases from 200 to 300 °C. Also, these results reveal that with higher temperatures, the formation of nanocrystals occurs at the surface, as observed in the samples annealed at 250 and 300 °C.

In order to know the active layer thickness, profilometer measurements were done. The thickness was measured in 5 different zones of the samples to get a median value since these layers are not highly uniform due to spin-coating. The thicknesses obtained were [22.5 ± 8.2] and [35 ± 4.1] nm for the samples annealed at 200 and 250 °C, respectively. It was not possible to obtain the thickness of the sample annealed at 300 °C due to the high surface roughness, as observed in the AFM image (Figure 2e).

### 3.3 MoO_3_ Memristor Characterization

The devices produced with different annealing temperatures were electrically analyzed to study their performance. The pristine state obtained at each temperature shows a symmetric *I*–*V* curve behavior in the range of [−0.5 to +0.5 V], as depicted in Figure S3. However, the device annealed at 250 °C is more resistive compared to the others. The device annealed at 200 °C is less resistive than that at 250 °C, likely related to the lower thickness. The device annealed at 300 °C is also less resistive than that at 250 °C. This likely indicates the existence of shunting paths (e.g., along grain boundaries) related to the formation of nanocrystallinity, observable by AFM. To activate the RS behavior, a positive voltage of 2 V was applied, followed by another sweep of 1 V with the same polarity, as demonstrated in Figure S4. This step was performed with a current compliance of 10 mA to prevent the breakdown. After the filament formation, the tests were done with the limiting current compliance of the equipment (0.1 A).

The forming characteristics followed by a reset at the same polarity are a typical property of thermochemical variant memristor devices. However, here, bipolar resistive switching with the set process at negative polarity and the reset process at positive polarity is the most stable RS property. Therefore, this two-step forming is assumed as the process of curing the thin film into the establishment of a resistive switching template. As reported in ref (25), there is an interplay between a typical redox reaction related to the TCM and the VCM. This means that depending on the dominance of the thermochemical/electrochemical redox, the VCM/TCM switching is observed. Sometimes, RS properties coexist in one cell depending on the current compliance or the presence of cation movements.^26^

**Figure 3:**
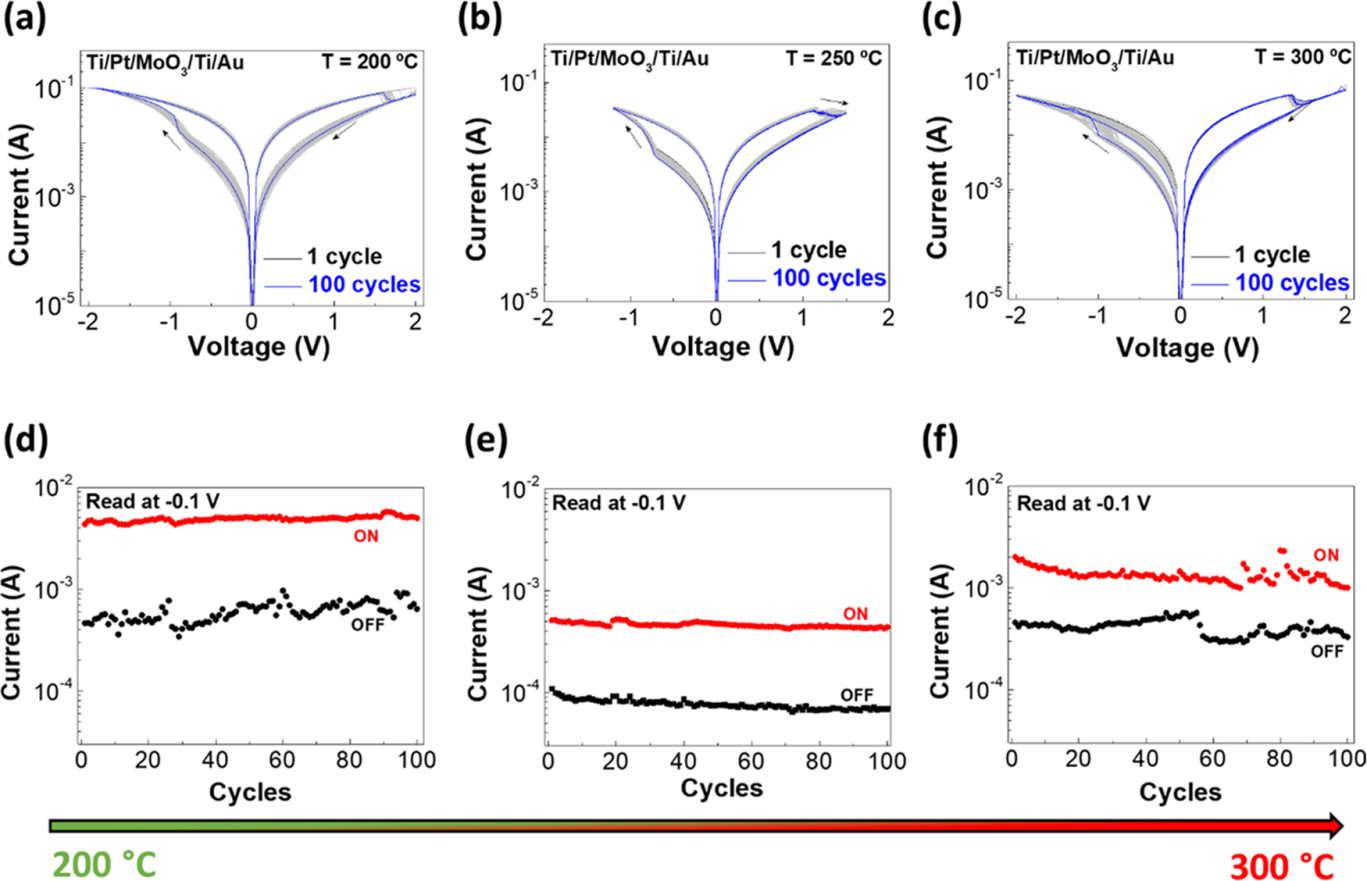
Endurance tests of the MoO_3_ memristors annealed at different temperatures: (a) 200, (b) 250, and (c) 300 °C, respectively. On/off ratio of each device at (d) 200, (e) 250, and (f) 300 °C.

Endurance tests were performed on the devices annealed at 200, 250, and 300 °C, as depicted in Figure 3a–c, respectively. All of the devices show a bipolar resistive switching behavior with a gradual set on negative polarity and an abrupt reset on positive polarity. Although all of the memristors reveal good endurance, devices annealed at 200 and 300 °C show high variability during cycles, as demonstrated in the corresponding Figure 3d,f by the on/off ratios. It is possible to observe that the most stable device during endurance tests is the memristor annealed at 250 °C (Figure 3e). Although the difference between each annealing temperature is small, it interferes right away with the stability of the device. As observed in TG-DSC and XPS results, 200 °C is a temperature which is not high enough to fully convert the precursors to MoO_3_, which is likely the cause for the state variations, as shown in Figure 3d. For the case of 300 °C, the high film roughness explains the endurance failure.

**Figure 4:**
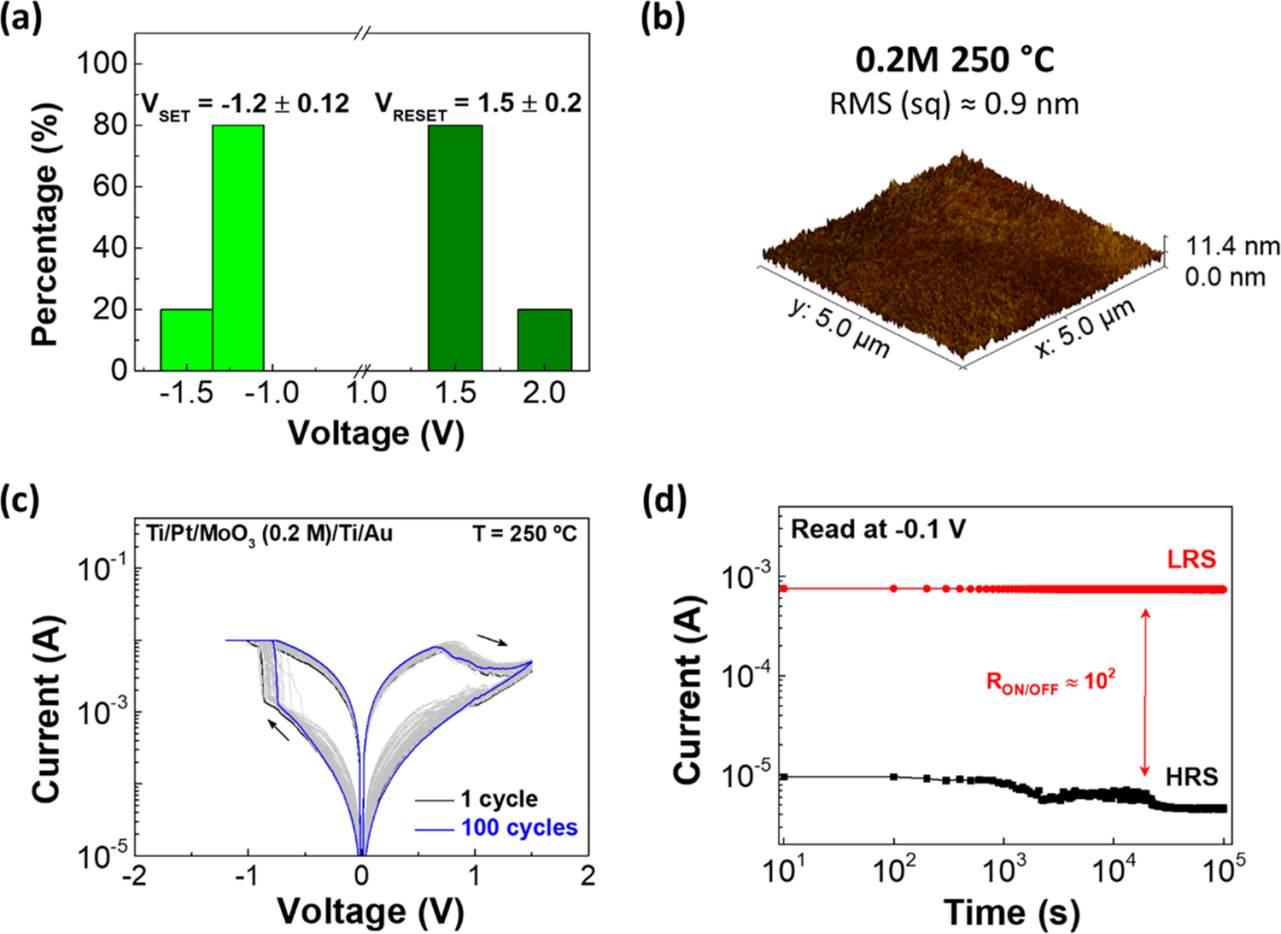
(a) Device-to-device variation of set and reset voltages in the sample with devices annealed at 250 °C. (b) AFM 3D image of the surface roughness of the sample MoO_3_ (0.2 M) annealed at 250 °C. (c) *I*–*V* curves obtained from endurance tests on devices with an active layer of MoO_3_ with 0.2 M. (d) Retention characteristics with read at −0.1 V during 10^5^ s for the HRS (black) and the LRS (red) of the same device.

Regarding these results, a retention test was performed on the device, as presented in Figure 3b,e. Figure S5 reveals the retention characteristics over time of the memristor in ambient air of up to 1 × 10^5^ s. Despite a decrease in its current over time in the HRS, the memristor exhibits no significant signs of degradation, which indicates that the device is stable over a long period of time and nonvolatile. The HRS current decrease during the retention test has been observed for other memristors deposited from solution.^27^ Electrocuring of residual precursor species may be the cause for the HRS current decrease. It is also important to study the reproducibility of these devices since the applications of memristors imply the use of hundreds or even thousands of devices at the same time.^28^ As shown in Figure 4a, it is possible to observe the device reproducibility of five devices annealed at 250 °C. The analysis demonstrates that the set and reset biases exhibit minimal deviations, with mean values of [−1.2 ± 0.12] and [1.5 ± 0.2], respectively. This indicates a consistent behavior across the devices with a uniform layer.

Since AFM analysis revealed that a higher annealing temperature enhances the roughness of the films, memristors were tested with only one layer by using a higher molar concentration of MoO_3_. With one deposited layer, it is possible to reduce the interfaces and artifacts that might affect the electrical performance of the memristor. So, a sample consisting of a single layer of MoO_3_ with a concentration of 0.2 M was produced at a temperature of 250 °C. Figure 4b reveals the surface roughness of this sample, an image obtained from AFM measurements. As shown in the figure, the surface is smoother when compared to the sample (0.1 M) annealed at the same temperature.

XPS analysis was performed in this sample and is depicted in Figure S6. The results obtained are identical with the data related to the sample annealed at the same temperature with 0.1 M. Cross-sectional SEM was done to understand the thickness of the active layer at 0.2 M, which is near 60 nm, as observed in Figure S7. Profilometer measurements performed as described before corroborate with the cross-sectional SEM images with a median thickness value of [58 ± 9] nm.

Regarding electrical characterization, endurance tests were made and they revealed a similar behavior compared to the previous memristors tested, as depicted in Figure 4b. However, the device has a higher on/off ratio (≈10^2^) as observed in the retention test, as shown in Figure 4c.

Hence, the usage of a single layer with a higher concentration does not have a significant effect on the memristor properties compared to the two layers with a lower concentration. This shows the robustness of the fabrication process and promotes an easier transition from spin-coating to printing deposition techniques.

### 3.4 Discussion of the Switching Mechanism

MoO_3_ is easily reduced to lower oxidation states.^29^ This reduction turns the material more conductive.^30^ The existence of lower oxidation states, including metallic Mo, in the MoO_3_ layer close to the interface to the Ti/Au top contact can be observed in Figure S8. This suggests that molybdenum oxide is asymmetric, with lower oxidation states predominantly close to the top electrode interface.

Based on this evidence, a resistive switching mechanism is proposed, which involves filaments of reduced Mo species. During the device electroforming in positive polarity, filaments of reduced Mo species extend toward the Pt bottom contact. In the literature, protons have been discussed as migrating species in memristors, which also lead to the reduction of Mo^6+^.^31^ The subsequent reset occurs in positive polarity as well. A possible explanation is the well-known oxygen exchange through platinum electrodes.^32,33^ Oxygen from the bottom interface reoxidizes molybdenum. During the set operation in negative polarity, oxygen is then incorporated again into platinum.

## 4 Conclusions

Solution-based MoO_3_ memristors were successfully fabricated and tested using an ink recipe based on water as a solvent for a more environmentally friendly approach. Structural analysis revealed that the annealing temperature significantly influenced the crystalline structure of the MoO_3_ thin films, showing amorphous characteristics below 300 °C. XPS analysis confirmed the presence of predominantly the Mo^6+^ oxidation state in samples annealed at different temperatures.

The memristors annealed at 250 °C exhibited a higher stability when compared with both lower and higher annealing temperatures, lower variability, and a retention time of 10^5^ s under ambient air conditions, indicating their nonvolatile behavior. One layered device with a higher concentration annealed at 250 °C revealed a higher on/off ratio and lower variability.

The excellent device reproducibility and robustness toward process variations in terms of the concentration and number of layers are crucial for scalability into large-scale production.

## Supporting Information Available

Tauc’s plot from the transmittance spectra; secondary edge spectrum from MoO_3_; atomic concentrations of Mo, O, C, and S; pristine states; electrical response before RS behavior; retention test of the sample annealed at 250 °C (0.1 M); XPS analysis of the 0.2 M sample; cross-sectional SEM image; fitted Mo 3d emission (PDF)

## References

[ref1] Patil A. R.; Dongale T. D.; Kamat R. K.; Rajpure K. Y. Binary Metal Oxide-Based Resistive Switching Memory Devices: A Status Review. Mater. Today Commun. 2023, 34, 105356 10.1016/j.mtcomm.2023.105356.

[ref2] Panda D.; Dhar A.; Ray S. K. Nonvolatile Memristive Switching Characteristics of TiO 2 Films Embedded with Nickel Nanocrystals. IEEE Trans. Nanotechnol. 2012, 11 (1), 51–55. 10.1109/TNANO.2011.2132142.

[ref3] Chen W.; Song L.; Wang S.; Zhang Z.; Wang G.; Hu G.; Gao S. Essential Characteristics of Memristors for Neuromorphic Computing. Adv. Electron. Mater. 2023, 9 (2), 2200833 10.1002/AELM.202200833.

[ref4] Memristors Market Size & Share Analysis - Industry Research Report - Growth Trends. https://www.mordorintelligence.com/industry-reports/memristor-market. (accessed May 28, 2023).

[ref5] Rajasekaran S.; Simanjuntak F. M.; Chandrasekaran S.; Panda D.; Saleem A.; Tseng T. Y. Flexible Ta2O5/WO3-Based Memristor Synapse for Wearable and Neuromorphic Applications. IEEE Electron Device Lett. 2022, 43 (1), 9–12. 10.1109/LED.2021.3127489.

[ref6] Panda D.; Hui Y. F.; Tseng T. Y. Diffusion Limiting Layer Induced Tantalum Oxide Based Memristor as Nociceptor. Mater. Today Electron. 2023, 3, 100031 10.1016/j.mtelec.2023.100031.

[ref7] Carlos E.; Branquinho R.; Martins R.; Kiazadeh A.; Fortunato E. Recent Progress in Solution-Based Metal Oxide Resistive Switching Devices. Adv. Mater. 2020, 2004328 10.1002/adma.202004328. 33314334

[ref8] Advances in Non-Volatile Memory and Storage Technology; Nishi Y.; Magyari-Kope B., Eds.; Elsevier, 2019.

[ref9] Arita M.; Kaji H.; Fujii T.; Takahashi Y. Resistance Switching Properties of Molybdenum Oxide Films. Thin Solid Films 2012, 520 (14), 4762–4767. 10.1016/j.tsf.2011.10.174.

[ref10] Avani A. V.; Anila E. I. Recent Advances of MoO3 Based Materials in Energy Catalysis: Applications in Hydrogen Evolution and Oxygen Evolution Reactions. Int. J. Hydrogen Energy 2022, 47 (47), 20475–20493. 10.1016/j.ijhydene.2022.04.252.

[ref11] Malik R.; Joshi N.; Tomer V. K. Advances in the Designs and Mechanisms of MoO 3 Nanostructures for Gas Sensors: A Holistic Review. Mater. Adv. 2021, 2 (13), 4190–4227. 10.1039/D1MA00374G.

[ref12] Gong Y.; Dong Y.; Zhao B.; Yu R.; Hu S.; Tan Z. Diverse Applications of MoO3 for High Performance Organic Photovoltaics: Fundamentals, Processes and Optimization Strategies. J. Mater. Chem. A 2020, 8 (3), 978–1009. 10.1039/C9TA12005J.

[ref13] Rasool A.; Amiruddin R.; Mohamed I. R.; Kumar M. C. S. Fabrication and Characterization of Resistive Random Access Memory (ReRAM) Devices Using Molybdenum Trioxide (MoO3) as Switching Layer. Superlattices Microstruct. 2020, 147, 106682 10.1016/j.spmi.2020.106682.

[ref14] Rahman F.; Ahmed T.; Walia S.; Mayes E.; Sriram S.; Bhaskaran M.; Balendhran S. Reversible Resistive Switching Behaviour in CVD Grown, Large Area MoO X. Nanoscale 2018, 10 (42), 19711–19719. 10.1039/C8NR04407D. 30141809

[ref15] Carlos E.; Kiazadeh A.; Deuermeier J.; Branquinho R.; Martins R.; Fortunato E. Critical Role of a Double-Layer Configuration in Solution-Based Unipolar Resistive Switching Memories. Nanotechnology 2018, 29 (34), 345206 10.1088/1361-6528/aac9fb. 29863489

[ref16] Carlos E.; Branquinho R.; Kiazadeh A.; Martins J.; Barquinha P.; Martins R.; Fortunato E. Boosting Electrical Performance of High-κ Nanomultilayer Dielectrics and Electronic Devices by Combining Solution Combustion Synthesis and UV Irradiation. ACS Appl. Mater. Interfaces 2017, 9 (46), 40428–40437. 10.1021/acsami.7b11752. 29090904

[ref17] Tan Z. H.; Yin X. B.; Guo X. One-Dimensional Memristive Device Based on MoO3 Nanobelt. Appl. Phys. Lett. 2015, 106 (2), 023503 10.1063/1.4906110.

[ref18] Du H.; Chen J.; Tu M.; Luo S.; Li S.; Yuan S.; Gong T.; Huang W.; Jie W.; Hao J. Transition from Nonvolatile Bipolar Memory Switching to Bidirectional Threshold Switching in Layered MoO 3 Nanobelts. J. Mater. Chem. C 2019, 7 (39), 12160–12169. 10.1039/C9TC03842F.

[ref19] Zhou G.; Wu J.; Wang L.; Sun B.; Ren Z.; Xu C.; Yao Y.; Liao L.; Wang G.; Zheng S.; Mazumder P.; Duan S.; Song Q. Evolution Map of the Memristor: From Pure Capacitive State to Resistive Switching State. Nanoscale 2019, 11 (37), 17222–17229. 10.1039/C9NR05550A. 31531487

[ref20] Bharathi M.; Balraj B.; Sivakumar C.; Wang Z.; Shuai J.; Ho M. S.; Guo D. Effect of Ag Doping on Bipolar Switching Operation in Molybdenum Trioxide (MoO3) Nanostructures for Non-Volatile Memory. J. Alloys Compd. 2021, 862, 158035 10.1016/j.jallcom.2020.158035.

[ref21] Bessonov A. A.; Kirikova M. N.; Petukhov D. I.; Allen M.; Ryhänen T.; Bailey M. J. A. Layered Memristive and Memcapacitive Switches for Printable Electronics. Nat. Mater. 2015, 14 (2), 199–204. 10.1038/nmat4135. 25384168

[ref22] Kovács T. N.; Hunyadi D.; de Lucena A. L. A.; Szilágyi I. M. Thermal Decomposition of Ammonium Molybdates. J. Therm. Anal. Calorim. 2016, 124 (2), 1013–1021. 10.1007/s10973-015-5201-0.

[ref23] Carcia P. F.; McCarron E. M. Synthesis and Properties of Thin Film Polymorphs of Molybdenum Trioxide. Thin Solid Films 1987, 155 (1), 53–63. 10.1016/0040-6090(87)90452-4.

[ref24] Baltrusaitis J.; Mendoza-Sanchez B.; Fernandez V.; Veenstra R.; Dukstiene N.; Roberts A.; Fairley N. Generalized Molybdenum Oxide Surface Chemical State XPS Determination via Informed Amorphous Sample Model. Appl. Surf. Sci. 2015, 326, 151–161. 10.1016/j.apsusc.2014.11.077.

[ref25] Dittmann R.; Menzel S.; Waser R. Nanoionic Memristive Phenomena in Metal Oxides: The Valence Change Mechanism. Adv. Phys. 2021, 70 (2), 155–349. 10.1080/00018732.2022.2084006.

[ref26] Wedig A.; Luebben M.; Cho D. Y.; Moors M.; Skaja K.; Rana V.; Hasegawa T.; Adepalli K. K.; Yildiz B.; Waser R.; Valov I. Nanoscale Cation Motion in TaOx, HfOx and TiOx Memristive Systems. Nat. Nanotechnol. 2016, 11 (1), 67–74. 10.1038/nnano.2015.221. 26414197

[ref27] Rosa J.; Kiazadeh A.; Santos L.; Deuermeier J.; Martins R.; Gomes H. L.; Fortunato E. Memristors Using Solution-Based IGZO Nanoparticles. ACS Omega 2017, 2 (11), 8366–8372. 10.1021/acsomega.7b01167. 31457375 PMC6644988

[ref28] Martins R. A.; Carlos E.; Deuermeier J.; Pereira M. E.; Martins R.; Fortunato E.; Kiazadeh A. Emergent Solution Based IGZO Memristor towards Neuromorphic Applications. J. Mater. Chem. C 2022, 10 (6), 1991–1998. 10.1039/D1TC05465A. PMC9241358 35873858

[ref29] Liao X.; Jeong A. R.; Wilks R. G.; Wiesner S.; Rusu M.; Bär M. X-Ray Irradiation Induced Effects on the Chemical and Electronic Properties of MoO3 Thin Films. J. Electron Spectrosc. Relat. Phenom. 2016, 212, 50–55. 10.1016/j.elspec.2016.08.004.

[ref30] Kaiser F.; Schmidt M.; Grin Y.; Veremchuk I. Molybdenum Oxides MoO x: Spark-Plasma Synthesis and Thermoelectric Properties at Elevated Temperature. Chem. Mater. 2020, 32 (5), 2025–2035. 10.1021/acs.chemmater.9b05075.

[ref31] Ahn M.; Park Y.; Lee S. H.; Chae S.; Lee J.; Heron J. T.; Kioupakis E.; Lu W. D.; Phillips J. D. Memristors Based on (Zr, Hf, Nb, Ta, Mo, W) High-Entropy Oxides. Adv. Electron. Mater. 2021, 7 (5), 2001258 10.1002/aelm.202001258.

[ref32] Branca N. C.; Deuermeier J.; Martins J.; Carlos E.; Pereira M.; Martins R.; Fortunato E.; Kiazadeh A. 2D Resistive Switching Based on Amorphous Zinc–Tin Oxide Schottky Diodes. Adv. Electron. Mater. 2020, 6 (2), 1900958 10.1002/aelm.201900958.

[ref33] Heisig T.; Baeumer C.; Gries U. N.; Mueller M. P.; La Torre C.; Luebben M.; Raab N.; Du H.; Menzel S.; Mueller D. N.; Jia C.-L.; Mayer J.; Waser R.; Valov I.; De Souza R. A.; Dittmann R. Oxygen Exchange Processes between Oxide Memristive Devices and Water Molecules. Adv. Mater. 2018, 30 (29), 1800957 10.1002/ADMA.201800957. 29882270

