# Supplementary material for: Low-Temperature Solution-Based Molybdenum Oxide Memristors
Source: ACS Appl Eng Mater. 2024 Jan 24;2(2):298–304. doi: 10.1021/acsaenm.3c00535 (PMC10897879; doi:10.1021/acsaenm.3c00535)
Supplement: Supplementary file 1 — em3c00535_si_001.pdf [file em3c00535_si_001.pdf]

Electronic Supplementary Information (ESI)

Low-temperature solution-based molybdenum oxide memristors

Raquel Azevedo Martins, Emanuel Carlos, Asal Kiazadeh, Rodrigo Martins, and Jonas Deuermeier**

CENIMAT|i3N, Department of Materials Science, School of Science and Technology, NOVA University Lisbon and CEMOP/UNINOVA, 2829-516 Caparica, Portugal.

*E-mail: j.deuermeier@fct.unl.pt and e.carlos@fct.unl.pt (corresponding authors)

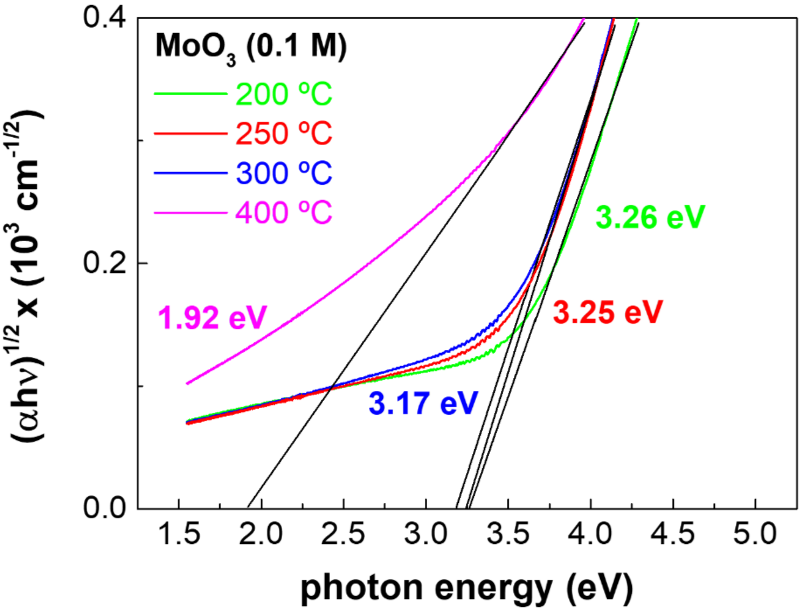

Figure S1. Tauc's plots to calculate each optical bandgap of MoO_3 thin films annealed with different temperatures.

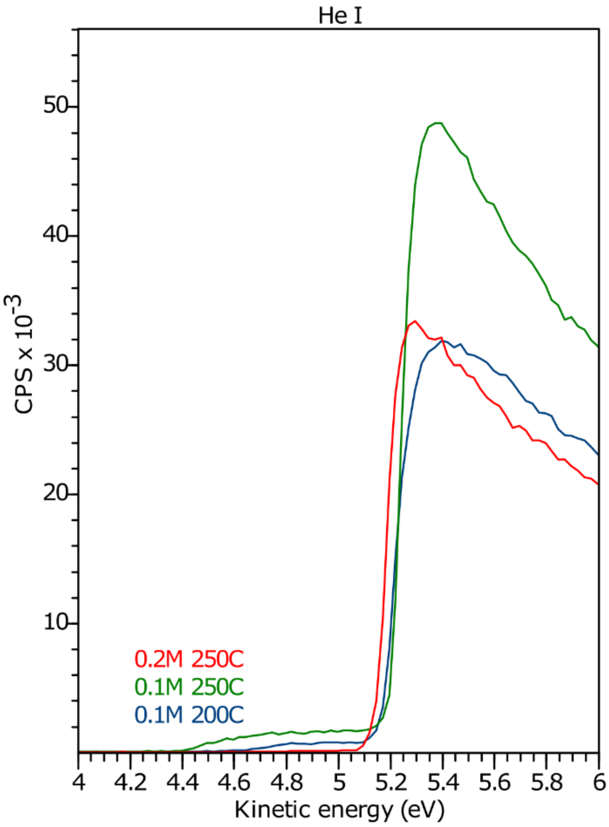

Figure S2. UPS Secondary electron edge with respect to kinetic energy from MoO_3 thin films deposited at 0.1M and annealed at 200 and 250 °C, as well as for 0.2M at 250 °C. Work function values were determined by linear extrapolation of the leading edge. The

data for the 0.1M, 300 °C sample was compromised by the appearance of a Pt signal, caused by the high roughness of the film.

At%	200 °C	250 °C	300 °C
**Mo 3d**	7.9	9.5	10.5
**O 1s**	66.9	76.6	81.5
**C 1s**	23.9	22.8	18.2
**S 2p**	1.2	0.6	0.3
**VB (eV)**	3.02	2.98	-
**WF (eV)**	5.17	5.20	-

Table S1. Atomic concentrations of Mo, O, C and S, as well as valence band maxima with respect to Fermi level and work functions from XPS. Note that a considerable amount of Pt emission was detected for the sample 0.1M, 300 °C. That indicates an incomplete coverage of the Pt layer, which is related to the high RMS roughness observed by AFM. This is the reason for not reporting VB and WF values for this sample.

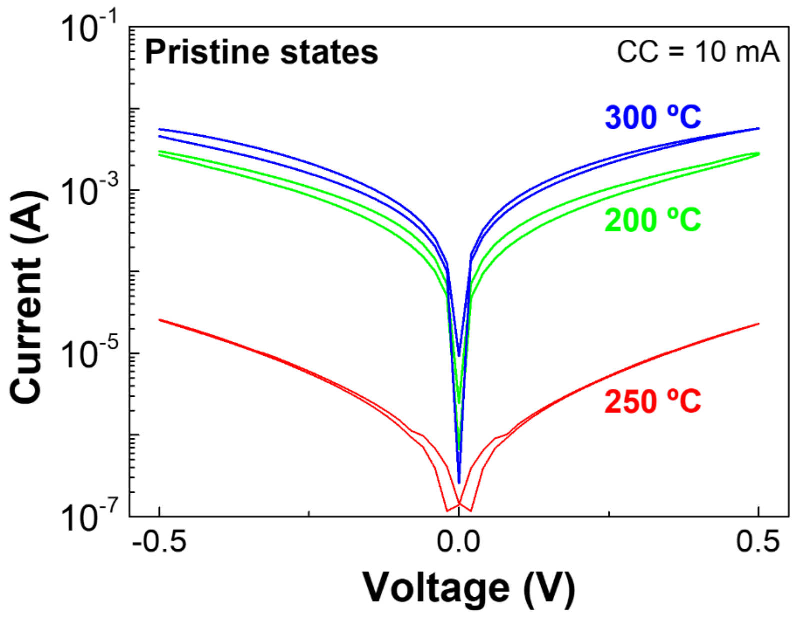

Figure S3. Pristine state of a memristor at each annealed temperature.

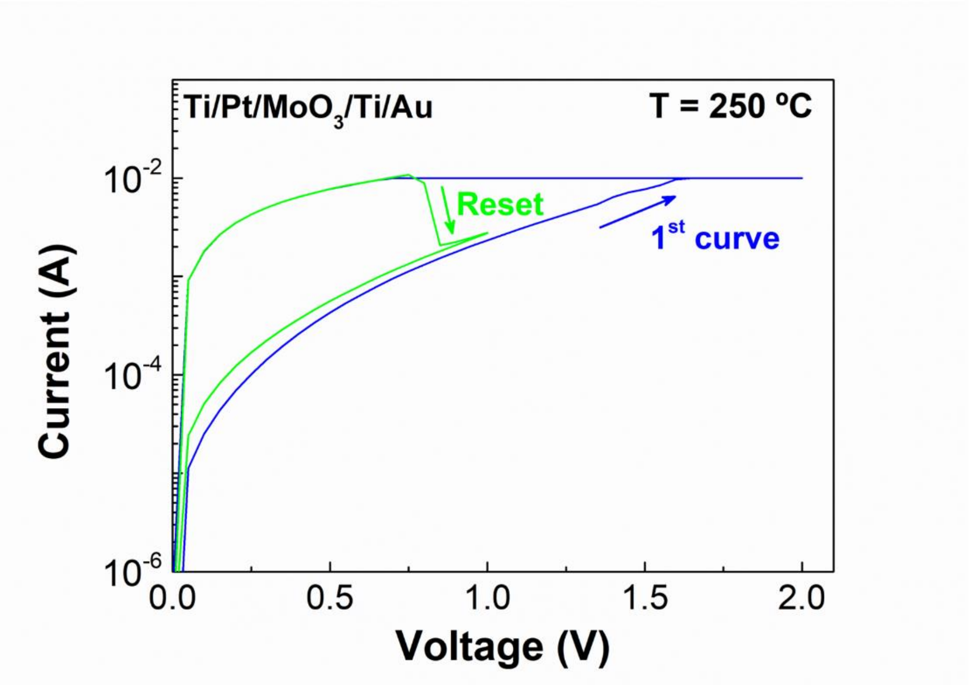

Figure S4. Typical response of the device before forming the conductive filament.

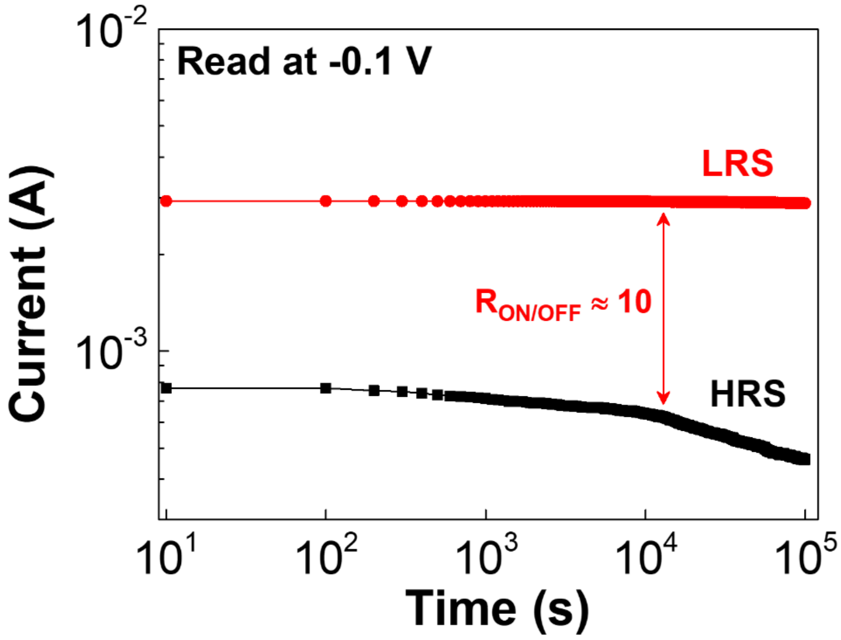

Figure S5. Retention time with read at - 0.1 V during 10^5 s for HRS (black) and LRS (red) of device shown in Figure 3b,e.

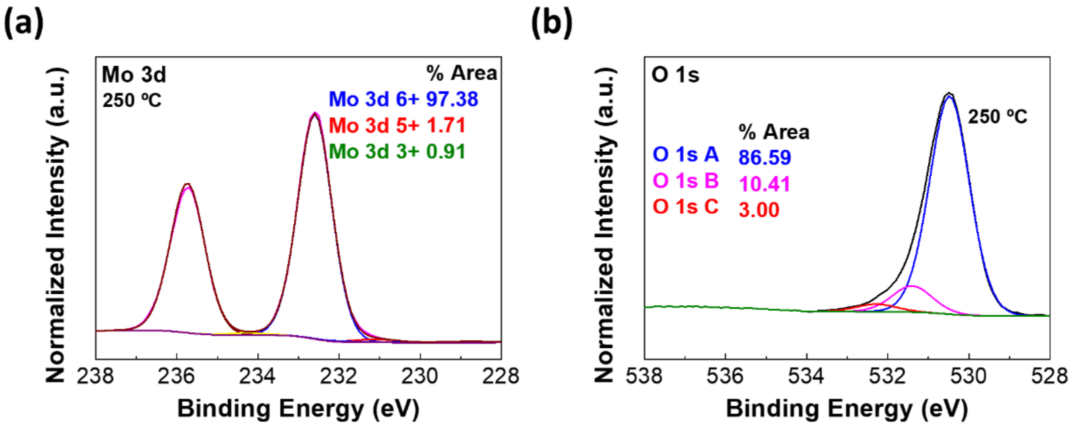

(c)

At%	250 °C
Mo 3d	9.3
O 1s	76.7
C 1s	22.4
S 2p	0.8
VB (eV)	3.01
WF (eV)	5.14

Figure S6. a) Mo 3d emission, b) O 1s emission of MoO3 and c) Atomic concentrations of Mo, O, C and S, as well as valence band maxima with respect to Fermi level and work functions of the 0.2 M sample.

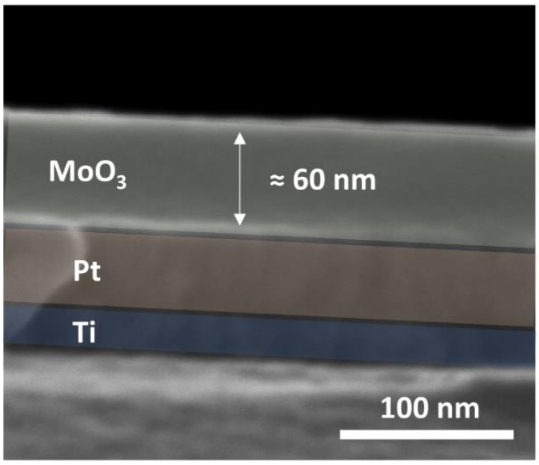

Figure S7. SEM cross section image of the sample MoO_3 (0.2M) on Ti/Pt annealed at 250 °C.

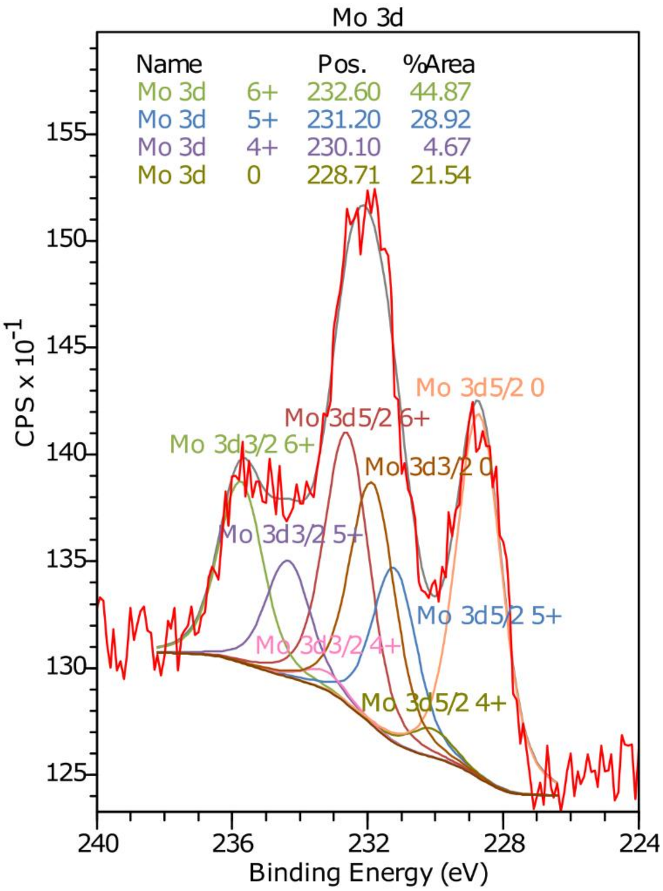

Figure S8. Fitted Mo 3d emission (according to [1]) of MoO_3 covered with around 6 nm Ti.

References

[1] J. Baltrusaitis *et al.*, "Generalized molybdenum oxide surface chemical state XPS determination via informed amorphous sample model," *Appl. Surf. Sci.*, vol. 326, pp. 151–161, Jan. 2015.
